# Fructose-1,6-Bisphosphate Protects Hippocampal Rat Slices from NMDA Excitotoxicity

**DOI:** 10.3390/ijms20092239

**Published:** 2019-05-07

**Authors:** Kamal M. Yakoub, Giacomo Lazzarino, Angela M. Amorini, Giuseppe Caruso, Concetta Scazzone, Marcello Ciaccio, Barbara Tavazzi, Giuseppe Lazzarino, Antonio Belli, Valentina Di Pietro

**Affiliations:** 1 Neurotrauma and Ophthalmology Research Group, School of Clinical and Experimental Medicine, College of Medical and Dental Sciences, University of Birmingham, Edgbaston, Birmingham B15 2TT, UK; k.yakoub@bham.ac.uk (K.M.Y.); a.belli@bham.ac.uk (A.B.); v.dipietro@bham.ac.uk (V.D.P.); 2 National Institute for Health Research Surgical Reconstruction and Microbiology Research Centre, Queen Elizabeth Hospital, Edgbaston, Birmingham B15 2TH, UK; 3 Institute of Biochemistry and Clinical Biochemistry, Catholic University of Rome, Largo F. Vito 1, 00168 Rome, Italy; giacomo.lazzarino@unicatt.it; 4 Fondazione Policlinico Universitario A. Gemelli IRCCS, Largo A. Gemelli 8, 00168 Rome, Italy; 5 Department of Biomedical and Biotechnological Sciences, Division of Medical Biochemistry, University of Catania, Viale A. Doria 6, 95125 Catania, Italy; amorini@unict.it; 6 Oasi Research Institute–IRCCS, Via Conte Ruggero 73, 94018 Troina (EN), Italy; forgiuseppecaruso@gmail.com; 7 Institute of Clinical Biochemistry, Clinical Molecular Medicine and Laboratory Medicine, Department of Biomedicine, Neurosciences and Advanced Diagnostics, Via del Vespro 129, 90127 Palermo, Italy; concetta.scazzone@unipa.it (C.S.); marcello.ciaccio@unipa.it (M.C.)

**Keywords:** fructose-1,6-bisphosphate, *N*-methyl-d-aspartate, excitotoxicity, energy metabolism, mitochondrial dysfunction, organotypic hippocampal brain slice cultures

## Abstract

Effects of fructose 1,6-bisphosphate (F-1,6-P2) towards *N*-methyl-d-aspartate NMDA excitotoxicity were evaluated in rat organotypic hippocampal brain slice cultures (OHSC) challenged for 3 h with 30 μM NMDA, followed by incubations (24, 48, and 72 h) without (controls) and with F-1,6-P2 (0.5, 1 or 1.5 mM). At each time, cell necrosis was determined by measuring LDH in the medium. Energy metabolism was evaluated by measuring ATP, GTP, ADP, AMP, and ATP catabolites (nucleosides and oxypurines) in deproteinized OHSC extracts. Gene expressions of phosphofructokinase, aldolase, and glyceraldehyde-3-phosphate dehydrogenase were also measured. F-1,6-P2 dose-dependently decreased NMDA excitotoxicity, abolishing cell necrosis at the highest concentration tested (1.5 mM). Additionally, F-1,6-P2 attenuated cell energy imbalance caused by NMDA, ameliorating the mitochondrial phosphorylating capacity (increase in ATP/ADP ratio) Metabolism normalization occurred when using 1.5 mM F-1,6-P2. Remarkable increase in expressions of phosphofructokinase, aldolase and glyceraldehyde-3-phosphate dehydrogenase (up to 25 times over the values of controls) was also observed. Since this phenomenon was recorded even in OHSC treated with F-1,6-P2 with no prior challenge with NMDA, it is highly conceivable that F-1,6-P2 can enter into intact cerebral cells producing significant benefits on energy metabolism. These effects are possibly mediated by changes occurring at the gene level, thus opening new perspectives for F-1,6-P2 application as a useful adjuvant to rescue mitochondrial metabolism of cerebral cells under stressing conditions.

## 1. Introduction

Brain damage caused by acute (ischemia, stroke, hypoxia, traumatic brain injury) and chronic neurological disorders (Alzheimer’s disease, Parkinson’s disease, multiple sclerosis) has some common biochemical features including ionic imbalance [1], cell energy failure [2] and increase in the release of neurotransmitters with inhibition of their reuptake [3]. This last phenomenon mainly involves glutamate, the most important excitotoxic neurotransmitter. Glutamate binds to ionotropic *N*-methyl-d-aspartate (NMDA), α-amino-3-hydroxy-5-methyl-4-isoxazolepropionic acid (AMPA), kainic acid rceptors, as well as to metabotropic receptors, promoting a major influx of calcium into neurons and astrocytes. Re-uptake by astrocytes is crucial to terminate glutamate signaling and to prevent the insurgence of the dangerous phenomenon known as glutamate excitotoxicity [4]. Using NMDA, which acts as an agonist for NMDA receptor, it is possible to mimic glutamate excitotoxicity in various experimental conditions [5,6].

Under pathological conditions, the glutamate-glutamine cycle involving neurons and astrocytes is impaired leading to glutamate excitotoxicity with massive calcium influx within mitochondria. Calcium overload initiates excitatory events involving free radical generation, triggering of apoptosis through processing caspase cleavage, opening of the mitochondrial permeability transition pore. This last event causes the depolarization of the mitochondrial inner membrane damaging the major role of mitochondria, i.e., the correct functioning of the electron transfer chain (ETC) coupled to oxidative phosphorylation (OXPHOS), and causing transient or permanent mitochondrial malfunction [2,5]. The consequences are a decreased efficiency in adenosine triphosphate (ATP) synthesis, with imbalance in ATP production and consumption and cell energy deficit. Therefore, decrease in the neuronal ATP levels contributes to exacerbate the imbalance in ionic homeostasis in brain cells and creates a vicious circle linking glutamate excitotoxicity, mitochondrial activity and cell energy metabolism [7,8]. Additionally, malfunctioning mitochondria cause increased production of reactive oxygen and nitrogen species (ROS and RNS), leading to the insurgence of oxidative/nitrosative stress [9], and release of proapoptotic factors, with consequent activation of caspases and the intrinsic apoptotic pathway [10].

Therefore, glutamate excitotoxicity has been considered since long time a strategic target for therapeutic interventions in brain insults of different etiology [11]. Despite positive results obtained in cellular and animal models targeting NMDA and AMPA receptors with different promising drugs [12,13], the subsequent clinical trials failed to improve outcome of the patients [14,15] so that effective pharmacologic treatments to decrease glutamate excitotoxicity in humans are still lacking.

Fructose-1,6-bisphosphate (F-1,6-P2) is a six carbon monosaccharide, found in both prokaryotes and eukaryotes, and produced in the third reaction of glycolysis through the activity of phosphofructokinase I. In the last decades, exogenous administration of F-1,6-P2, in concentrations (1.5–10 mM) largely exceeding its intracellular concentration (1–5 μM) [16,17], has been used in a variety of pathological conditions in both preclinical and clinical studies [18]. Particularly, F-1,6-P2 improved myocardial energy metabolism [19] and decreased oxidative stress in experimental models of anoxia and re-oxygenation [20], reduced post-ischemic ventricular dysfunctions [21], improved outcome after circulatory arrest in pigs [22].

Clinically, F-1,6-P2 improved hemodynamic in patients with altered ventricular functions [23] and protected myocardium during coronary bypass surgery [24]. In models of brain injury, F-1,6-P2 protected neuronal integrity after circulatory arrest in pigs [25], preserved cerebral energy metabolism after brain ischemia [26], protected hippocampal neurons by repeated febrile convulsions [27], preserved glucose metabolism and decreased oxidative stress caused by experimental sepsis [28]. It is worth recalling that in different experimental conditions of hypoxia/ischemia F-1,6-P2 administration did not show significant beneficial effects on high energy phosphate metabolism [29,30].

Organotypic slice cultures represent a valid experimental model, maintaining neuronal maturation and plasticity, useful to evaluate experimental insults and drug treatments that can be followed for various days in vitro [31]. Using this model, toxicity of the Aβ oligomer 1–40, characteristic of the Alzheimer’s disease, has been evaluated in mixed neuronal-glial cerebellar cultures and organotypic cerebellar cultures [32]. Organotypic hippocampal slice cultures (OHSC) specifically uses hippocampal slices, mainly obtained from neonatal rats, to study the biochemical, morphological and functional characteristics of both neurons and glial cells. This model is suitable to study neurotoxicity, neuroprotection, neuronal networks or tumor invasion [33], and even to effectively mimic cellular damages induced by traumatic brain injury [34].

With the aim to clarify the effects of exogenous F-1,6-P2, in this study we tested the benefits of increasing concentrations of F-1,6-P2 on NMDA excitotoxicity induced in rat OHSC by measuring release of lactate dehydrogenase (LDH) in the medium and concentrations of various metabolites related to energy metabolism (ATP, GTP, ADP, AMP, oxypurines, nucleosides, lactate). The expression of the genes encoding for 6-phosphofructo-1-kinase (PFKL), aldolase (ALDOC) and glyceraldehyde-3-phosphate dehydrogenase (GAPDH), were also considered.

## 2. Results

### 2.1. NMDA Cytotoxicity and Protection by F-1,6-P2

**Figure 1:**
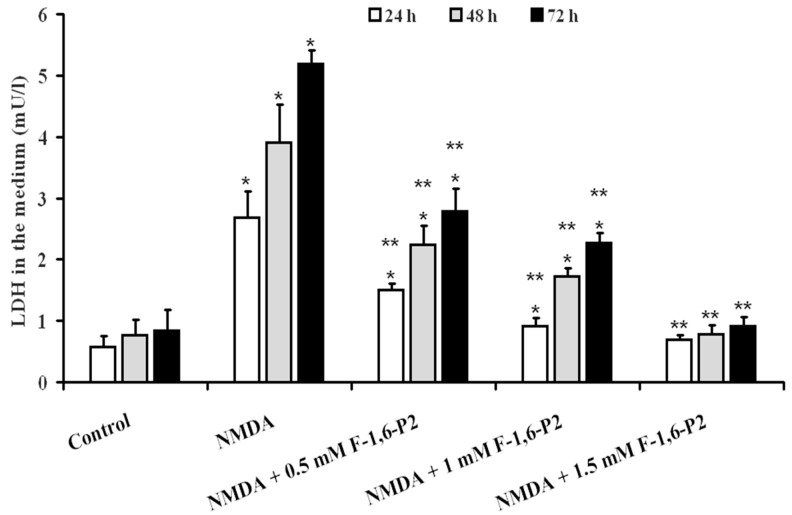
Protective effects of F-1,6-P2 on cell necrosis in NMDA-treated OHSC. OHSC were challenged with 30 μM NMDA for 3 h and then incubated without and with increasing concentrations of F-1,6-P2 (0.5, 1 and 1.5 mM) for different times (24, 48 and 72 h). Control OHSC were incubated for the same time intervals, with no prior NMDA treatment or F-1,6-P2 addition. Cell necrosis was deducted by the amount of LDH released in the culture medium and expressed as mU of enzyme activity/L of medium (1 U = 1 μmol/min of substrate consumed). Histograms are the mean of 6 different OHSC preparations. Standard deviations are represented by vertical bars. * Significantly different from corresponding time of control OHSC, *p* < 0.001. ** Significantly different from corresponding time of NMDA-challenged OHSC with no F-1,6-P2 supplementation, *p* < 0.001.

The cytotoxic effects of NMDA on OHSC were clearly evidenced by the time-dependent increase of LDH in the medium (Figure 1). At 24, 48 and 72 h after the 3 h challenge with 30 µM NMDA, OHSC had LDH values were 4.7, 5.1 and 6.2 times higher than the values determined in the medium of control OHSC at the same time points (*p* < 0.001). The supplementation to the culture medium of NMDA-treated OHSC with 0.5, 1 or 1.5 mM F-1,6-P2 provoked a dose-dependent beneficial effect, as indicated by the decreased release of LDH (Figure 1). All samples receiving F-1,6-P2 in the recovery phase, for 24, 48 and 72 h post challenge with 30 µM NMDA, showed lower LDH values in the medium at any time point, when compared to the corresponding times of OHSC with no supplementation (*p* < 0.001). It is worth noting that 1.5 mM F-1,6-P2 completely abolished the cytotoxic effects of NMDA, so that LDH values in the medium of these cultures were not significantly different from those found in controls at the corresponding time points (Figure 1).

### 2.2. NMDA-Induced Imbalance of OHSC Energy Metabolism and Protection by F-1,6-P2 

**Figure 2:**
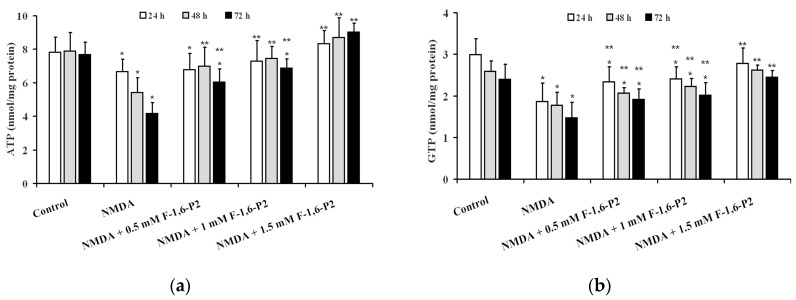
Effects of F-1,6-P2 on the recovery of high energy phosphate concentrations in NMDA-treated OHSC. OHSC were challenged with 30 μM NMDA for 3 h and then incubated without and with increasing concentrations of F-1,6-P2 (0.5, 1 and 1.5 mM) for different times (24, 48 and 72 h). Control OHSC were incubated for the same time intervals, with no prior NMDA treatment or F-1,6-P2 addition. ATP (**a**) and GTP (**b**) were determined by HPLC in deproteinized cell extracts. Histograms are the mean of 6 different OHSC preparations. Standard deviations are represented by vertical bars. * Significantly different from corresponding time of control OHSC, *p* < 0.001. ** Significantly different from corresponding time of NMDA-challenged OHSC with no F-1,6-P2 supplementation, *p* < 0.001.

Prolonged recovery following incubation of OHSC with 30 µM NMDA for 3 h caused a time-dependent decline in the cell energy state. As shown in Figure 2, ATP (panel A) and GTP (panel B) concentrations in NMDA-pretreated OHSC at 24, 48 and 72 h incubation were 15%, 31% and 46%, and 37%, 32% and 39% lower, respectively, than corresponding values detected in controls (*p* < 0.001). The addition during the recovery phase of increasing concentrations of F-1,6-P2 (0.5, 1 or 1.5 mM), to the medium of 30 μM NMDA-pretreated OHSC, significantly protected cell energy metabolism. Levels of both high energy phosphates (ATP and GTP) in OHSC receiving F-1,6-P2 were significantly higher than the values recorded in OHSC with no supplement during recovery. It is particularly relevant that the beneficial effects produced by the presence of 1.5 mM F-1,6-P2, during recovery of OHSC after challenging with 30 μM NMDA, were capable to allow measuring values of ATP and GTP that were not different from those found in control OHSC.

**Figure 3:**
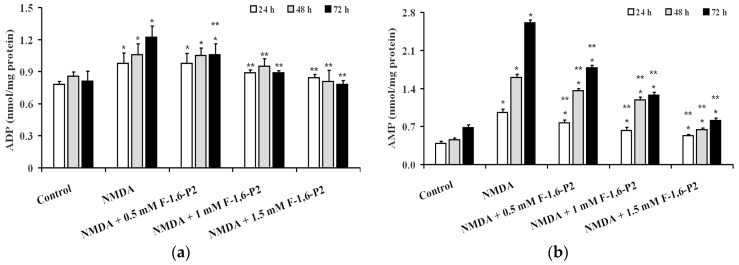
Effects of F-1,6-P2 on the imbalance of adenine nucleotide metabolism in NMDA-treated OHSC. OHSC were challenged with 30 μM NMDA for 3 h and then incubated without and with increasing concentrations of F-1,6-P2 (0.5, 1 and 1.5 mM) for different times (24, 48 and 72 h). Control OHSC were incubated for the same time intervals, with no prior NMDA treatment or F-1,6-P2 addition. ADP (**a**) and AMP (**b**) were determined by HPLC in deproteinized cell extracts. Histograms are the mean of 6 different OHSC preparations. Standard deviations are represented by vertical bars. * Significantly different from corresponding time of control OHSC, *p* < 0.001. ** Significantly different from corresponding time of NMDA-challenged OHSC with no F-1,6-P2 supplementation, *p* < 0.001.

The consequence of OHSC energy metabolism imbalance, caused by 30 μM NMDA, was particularly evident when considering variations of AMP during the recovery phase, rather than when observing ADP changes (Figure 3). At 24, 48 and 72 h post NMDA challenge, ADP and AMP were 1.2, 1.2 and 1.5, and 2.5, 3.5 and 3.8 times higher than the values determined at corresponding times in control OHSC (*p* < 0.001). Addition of 1 and 1.5 mM F-1,6-P2 completely abolished the negative effect on ADP concentrations caused by pre-treatment with 30 μM NMDA (*p* < 0.001 compared to corresponding times of OHSC pretreated with NMDA; not significant compared to corresponding times of control OHSC). F-1,6-P2 was less effective in normalizing AMP concentrations. Even at the highest dose tested (1.5 mM), AMP in OHSC (pretreated with 30 μM NMDA) receiving F-1,6-P2 during recovery was significantly lower than values found at corresponding times in OHSC (pretreated with 30 μM NMDA) with no addition during recovery (*p* < 0.001), but also significantly higher than concentrations measured in control OHSC (*p* < 0.001).

**Figure 4:**
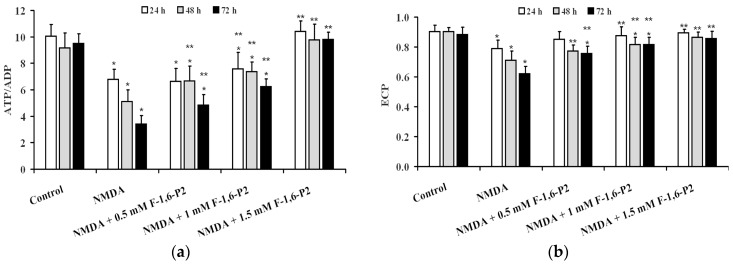
Effects of F-1,6-P2 on energy metabolism derangement in NMDA-treated OHSC. OHSC were challenged with 30 μM NMDA for 3 h and then incubated without and with increasing concentrations of F-1,6-P2 (0.5, 1 and 1.5 mM) for different times (24, 48 and 72 h). Control OHSC were incubated for the same time intervals, with no prior NMDA treatment or F-1,6-P2 addition. ATP/ADP ratio (**a**) reflects the phosphorylating capacity of mitochondria. ECP (**b**) indicates the cell energy wellness, according to the equation: ECP = ATP + 1/2ADP/(ATP + ADP + AMP). Histograms are the mean of 6 different OHSC preparations. Standard deviations are represented by vertical bars. * Significantly different from corresponding time of control OHSC, *p* < 0.001. ** Significantly different from corresponding time of NMDA-challenged OHSC with no F-1,6-P2 supplementation, *p* < 0.001.

Data illustrated in Figure 4 indicate that using two indexes representing mitochondrial phosphorylating capacity (ATP/ADP ratio (Figure 4a) and cell energy wellness (ECP = energy charge potential = (ATP + 1/2ADP)/(ATP + ADP + AMP) (Figure 4b), it was possible to evidence that 3 h incubation with 30 μM NMDA caused long lasting negative effects on energy metabolism of OHSC cultures, that progressed by prolonging the time of recovery (24, 48 and 72 h). Dose-dependent ability to ameliorate both parameters was observed when supplementing 30 μM NMDA-pretreated OHSC with F-1,6-P2 0.5, 1 and 1.5 mM. At the highest dose tested (1.5 mM), OHSC receiving F-1,6-P2 had values of the ATP/ADP ratio and ECP not different from those calculated in control OHSC.

### 2.3. Effects of F-1,6-P2 on Adenine Nucleotide Catabolism and Lactate Production in OHSC Challenged with NMDA

**Figure 5:**
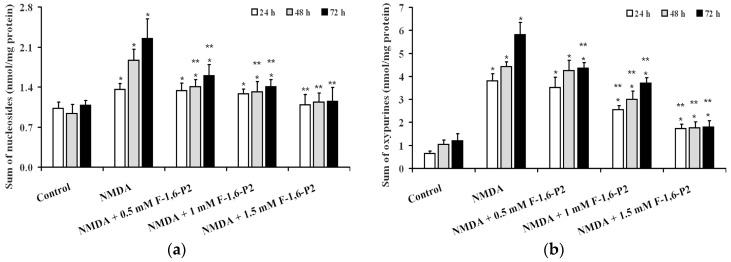
Effects of F-1,6-P2 on the imbalance of adenine nucleotide catabolism in NMDA-treated OHSC. OHSC were challenged with 30 μM NMDA for 3 h and then incubated without and with increasing concentrations of F-1,6-P2 (0.5, 1 and 1.5 mM) for different times (24, 48 and 72 h). Control OHSC were incubated for the same time intervals, with no prior NMDA treatment or F-1,6-P2 addition. Sum of nucleosides (**a**) = adenosine + inosine + guanosine. Sum of oxypurines (**b**) = hypoxanthine + xanthine + uric acid. Nucleosides and oxypurines were determined by HPLC in deproteinized cell extracts. Histograms are the mean of 6 different OHSC preparations. Standard deviations are represented by vertical bars. * Significantly different from corresponding time of control OHSC, *p* < 0.001. ** Significantly different from corresponding time of NMDA-challenged OHSC with no F-1,6-P2 supplementation, *p* < 0.001.

Quantification in the culture medium of products generating from ATP dephosphorylation (adenosine, inosine, hypoxanthine, xanthine and uric acid), evidenced the activation of the degradation pathway of adenine nucleotide catabolism in OHSC pretreated with 30 μM NMDA and then allowed to recover for 24, 48 and 72 h (Figure 5). Both sum of nucleosides (Figure 5a) and sum of oxypurines (Figure 5b) progressively increased during the time of recovery (*p* < 0.001 compared to corresponding times of control OHSC), with sum of oxypurines showing the most dramatic increases in the medium (5.8, 4.2 and 4.8 times at 24, 48 and 72 h, respectively; *p* < 0.001).

**Figure 6:**
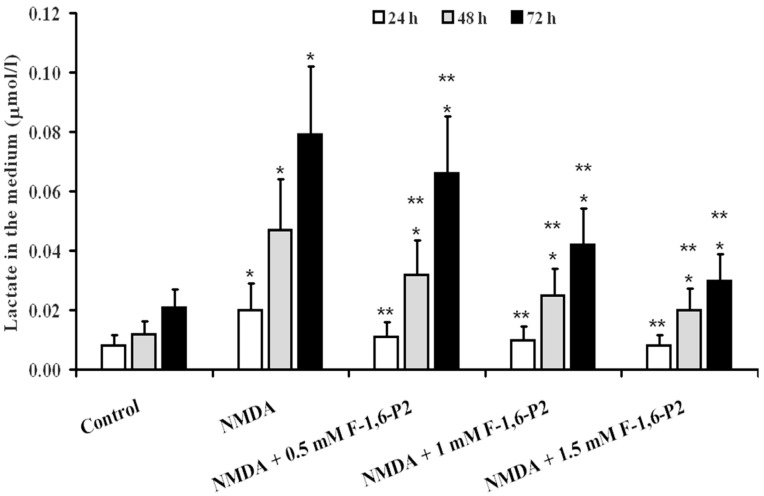
Effects of F-1,6-P2 on lactate production in NMDA-treated OHSC. OHSC were challenged with 30 μM NMDA for 3 h and then incubated without and with increasing concentrations of F-1,6-P2 (0.5, 1 and 1.5 mM) for different times (24, 48 and 72 h). Control OHSC were incubated for the same time intervals, with no prior NMDA treatment or F-1,6-P2 addition. Lactate was measured enzymatically in deproteinized cell extracts. * Significantly different from corresponding time of control OHSC, *p* < 0.001. ** Significantly different from corresponding time of NMDA-challenged OHSC with no F-1,6-P2 supplementation, *p* < 0.001.

The release of lactate in the medium of OHSC after challenge with 30 μM NMDA (Figure 6) increased by 2.5, 3.9 and 3.8 times, respectively, at 24, 48 and 72 h, compared to values measured at corresponding times in control OHSC (*p* < 0.001). Significantly lower values were found at any time point when F-1,6-P2, at any dose tested, was added to the medium of OHSC during the recovery phase (*p* < 0.001). However, at 48 and 72 h after NMDA challenge, cultures supplemented with F-1,6-P2 had lactate release in the medium higher than that measured in control OHSC (*p* < 0.001).

### 2.4. Expression of Genes Regulating the Synthesis of Glycolytic Enzymes Involved in F-1,6-P2 Metabolism

Under all experimental conditions, we determined the expression of genes controlling the synthesis of the isoform L of 6-phospho-1-fructokinase (PFKL), aldolase (brain gene = ALDOC) and glyceraldehyde-3-phospate dehydrogenase (GAPDH). These three enzymes are sequentially positioned along the glycolytic pathway providing to the synthesis of F-1,6-P2 from fructose-6-phosphate and ATP (PFKL), to its hydrolysis into glyceraldehyde-3-phospate and dihydroxyacetone-3-phosphate (aldolase) and to the oxidation and phosphorylation of glyceraldehyde-3-phospate into glycerate-1,3-bisphosphate (GAPDH). 

**Figure 7:**
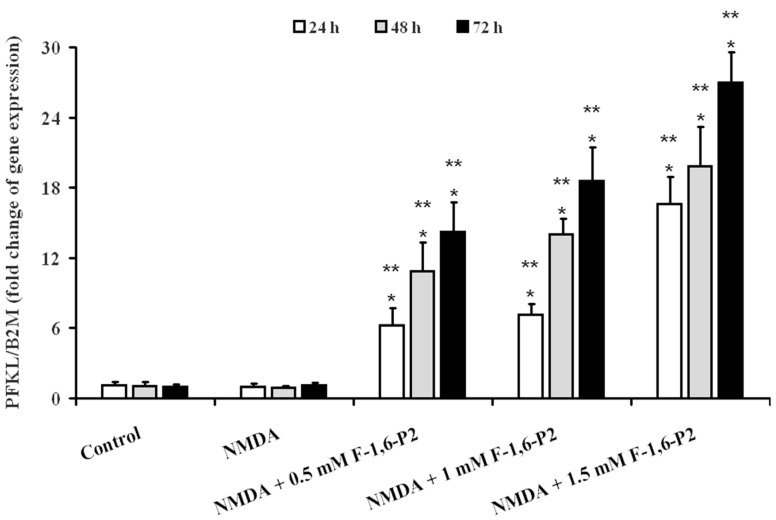
Effects of F-1,6-P2 on gene expression of 6-phospho-1-fructokinase (PFKL) in NMDA-treated OHSC. OHSC were challenged with 30 μM NMDA for 3 h and then incubated without and with increasing concentrations of F-1,6-P2 (0.5, 1 and 1.5 mM) for different times (24, 48 and 72 h). Control OHSC were incubated for the same time intervals, with no prior NMDA treatment or F-1,6-P2 addition. Semi-quantitative PFKL expression was determined in cell extracts, relatively to the housekeeping gene β-2-microglobulin (B2M). Histograms are the mean of 6 different OHSC preparations. Standard deviations are represented by vertical bars. * Significantly different from corresponding time of control OHSC, *p* < 0.001. ** Significantly different from corresponding time of NMDA-challenged OHSC with no F-1,6-P2 supplementation, *p* < 0.001.

Neither control OHSC nor OHSC pretreated with 30 μM NMDA and incubated for 24, 48 and 72 h of recovery showed any difference in the gene expression of PFKL, at any time point (Figure 7). 

When 0.5 mM F-1,6-P2 was added to the medium after challenge with 30 μM NMDA, PFKL expression at 24, 48 and 72 h increased by 6, 10 and 14 times, respectively, over the values of control OHSC at corresponding time points (*p* < 0.001). Using 1 mM F-1,6-P2 this increase was 7, 14 and 19 times at 24, 48 and 72 h, respectively (*p* < 0.001 compared to control OHSC). At the highest dose of F-1,6-P2 tested (1.5 mM), expression of PFKL at the same time points was 17, 20 and 27 times higher than the values recorded in control OHSC (*p* < 0.001).

**Figure 8:**
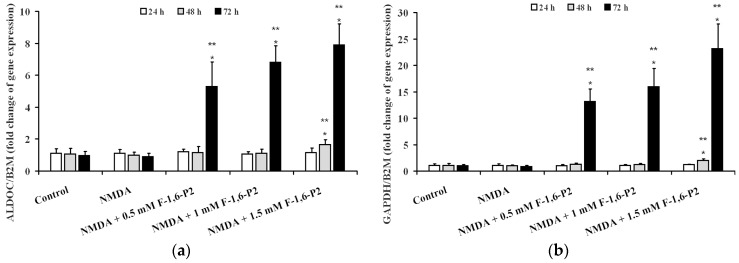
Effects of F-1,6-P2 on gene expression of aldolase (ALDOC) and glyceraldehyde-3-phosphate dehydrogenase (GAPDH) in NMDA-treated OHSC. OHSC were challenged with 30 μM NMDA for 3 h and then incubated without and with increasing concentrations of F-1,6-P2 (0.5, 1 and 1.5 mM) for different times (24, 48 and 72 h). Control OHSC were incubated for the same time intervals, with no prior NMDA treatment or F-1,6-P2 addition. Semi-quantitative ALDOC (**a**) and GAPDH (**b**) expressions were determined in cell extracts, relatively to the housekeeping gene β-2-microglobulin (B2M). Histograms are the mean of 6 different OHSC preparations. Standard deviations are represented by vertical bars. * Significantly different from corresponding time of control OHSC, *p* < 0.001. ** Significantly different from corresponding time of NMDA-challenged OHSC with no F-1,6-P2 supplementation, *p* < 0.001.

Differently, 0.5 and 1 mM F-1,6-P2 significantly affected ALDOC expression, after challenge with NMDA (Figure 8a), only after 72 h. Using these two dosages, expression of ALDOC increased by 5 and 7 times over the value measured in control OHSC (*p* < 0.001). When 1.5 mM F-1,6-P2 was added to the medium during the recovery phase, ALDOC increased 1.7 and 8 times at 48 and 72 h, respectively, in comparison with values recorded at corresponding times in control OHSC (*p* < 0.001). Similarly, 0.5 and 1 mM F-1,6-P2 produced 13 and 16 times increase, respectively, in GAPDH expression only after 72 h of recovery post challenge with NMDA (*p* < 0.001 compared to the same time of control OHSC) (Figure 8b). Again, the effect on this gene expression of NMDA pretreated OHSC supplemented with 1.5 mM F-1,6-P2 was significant either at 48 or at 72 h when 2 and 23 times higher values than those of control OHSC were recorded (*p* < 0.001).

In a different set of experiments, we evaluated the effects of the addition of increasing concentrations of F-1,6-P2 on the gene expressions of PFKL, ALDOC and GAPDH of OHSC with no previous challenge with NMDA. Data summarized in Table 1 showed that F-1,6-P2 caused the increase of the three genes with levels of overexpression slightly lower than those found when OHSC were previously challenged with 30 μM NMDA.

## 3. Discussion

The phenomenon of glutamate-mediated excitotoxicity plays a central role in acute and chronic neurodegenerations and represents a target for new therapeutic approaches in numerous pathological states, including cerebral ischemia, stroke, traumatic brain injury [35,36,37]. Organotypic hippocampal slice cultures represent a useful in vitro model with which to study not only the effects of excitotoxicity (triggered by the challenge with NMDA) on nervous cell metabolism and functions [38] but also to evaluate efficacy of drug treatments [39]. 

In the present study, treatment of OHSC with 30 μM NMDA for 3 h at 37 °C induced long lasting damages to nervous cells progressing during the whole observational time (72 h). Cell death increased by increasing the time of incubation and was accompanied by decrease in ATP concentration. Although part of this effect might be related to decrease in the number of living cells, it is worth underlining that dramatic decrease in the ATP/ADP ratio, which is considered a good indicator of the mitochondrial phosphorylating capacity [40], and remarkable reduction of the ECP, representing a measure of the energy wellness of the cell [41], were recorded. In addition, the derangement of energy metabolism in OHSC after treatment with NMDA was also evidenced by the almost three times and six times increase in AMP and oxypurines, respectively, strongly demonstrating activation of the adenine nucleotide degradation pathway depleting the cellular content of the high-energy phosphate compounds. In this context of sustained dysmetabolism, cerebral cell response was to increase the rate of glycolysis (increase in lactate efflux in the medium, Figure 6) without changing the expression either of the gene encoding for the key regulatory enzyme of glycolysis (PFKL) or of the genes encoding for the immediately following enzymes of the glycolytic pathway (ALDOC and GAPDH) (Figure 7 and Figure 8). This phenomenon, termed as hyperglycolysis, has been shown to occur following TBI [42] and has been associated to the sub-chronic phase following severe (but not mild) TBI [7].

The addition to the incubation medium of F-1,6-P2 following the induction of excitotoxicity had dose-dependent beneficial effects on cerebral cell survival and metabolism. Our results seem to inversely associate reduction of cell mortality with improvement in energy metabolism caused by F-1,6-P2. Particularly at the highest concentration tested, F-1,6-P2 restored ATP and GTP concentrations to values detected in control OHSC. ATP rescue was accompanied by high value of the ATP/ADP ratio and ECP, and decreased efflux of lactate in the medium (but still higher than control OHSC), thus indicating no mitochondrial dysfunction and normal cell energy state.

Results of the gene expression analysis clearly showed, for the first time to the best of our knowledge, that the gene encoding for 6-phosphofructo-1-kinase (PFKL) was time and dose-dependently increased by any dose of F-1,6-P2 tested. Additionally, at the longest time of incubation post NMDA challenge, a dose dependent increase in ADLOC and GAPDH was also observed. 

These results strongly suggest that F-1,6-P2, notwithstanding is a highly negatively charged molecule at physiological pH, can enter the cell in its intact form where it acts as a neuroprotector against NMDA excitotoxicity. While at physiological concentration the biochemical role of F-1,6-P2 as a substrate of aldolase and a positive allosteric modulator of 6-phosphofructo-1-kinase is well established [43], little is known either about the mechanisms involved in its cellular uptake or about the biochemical processes involved in its beneficial effects under conditions of cell sufferance. 

Using the isolated Langendorff-perfused rat heart, we have previously shown that exogenous F-1,6-P2 is actively metabolized by intact cardiomyocytes [44] and taken up through the involvement of the dicarboxylate transporter [45]. Since this transporter has been found in cerebral cells [46,47], it is conceivable hypothesizing that, even under the experimental conditions used in the present study, F-1,6-P2 might cross the nervous cell membranes involving the same transport mechanism. 

Although it has not been demonstrated whether F-1,6-P2 is a privileged, advantageous substrate for cell metabolism (its use in alternative to glucose would allow saving two ATP-dependent phosphorylating reactions, leading to a net production of 4 ATP moles/mole of F-1,6-P2 consumed, rather than 2 moles of ATP/mole of glucose consumed through glycolysis), several studies have shown that exogenous F-1,6-P2 at various dosage improves energy metabolism under conditions of cellular energetic crisis [19,20,21,22,23,24,25]. These effects, causing the increase in the concentrations of high-energy phosphates and of the cell phosphorylation potential [19,20,48], have been connected, in part, to a decrease in intracellular free calcium ions having positive influence on mitochondrial functions [49].

According to the results of the gene expressions of PFKL, ALDOC and GAPDH it is possible adding to the list of changes of cell metabolism/functions, induced by exogenous F-1,6-P2, this previously unknown and unexpected effect. If the increase in ALDOC and GAPDH can be interpreted as an adaptive mechanism, occurring only after relatively long time of incubation with F-1,6-P2 and after PFKL have remarkably increased, the augmentation of PFKL is certainly a direct consequence of the presence of increasing concentrations of F-1,6-P2 in the OHSC culture medium during the recovery phase, post NMDA challenge. Since these overexpressions were also measured in OHSC incubated for the same times with the same dosages of F-1,6-P2, but without prior challenge with NMDA, it is possible to exclude that uptake of F-1,6-P2 might take place only under conditions of altered cell membrane permeability, as it occurs after NMDA treatment [50]. 

It can be hypothesized that the overexpressions of the aforementioned genes result in a robust increase of the glycolytic flow. If occurring during mitochondrial dysfunction, higher glycolytic rates may be deleterious for cerebral cells and provoke hyperglycolysis [7,50]. Differently, when taking place with normal mitochondrial functions, higher glycolytic rates lead to improvement of cell energy state [7,51]. Furthermore, various studies underlined the roles of ALDOC and GAPDH in the cross-talk between neurons and astrocytes and in the physiopathological mechanisms regulating cerebral cell energy metabolism [52,53,54,55,56,57]. 

Although further studies in laboratory animals are needed, these results corroborate previous observations showing the beneficial effects of F-1,6-P2 on brain metabolism and functions under various stressing conditions [19,20,21,22,23,24,25,26]. The previously unreported effects of this drug on the expressions of genes encoding for key glycolytic enzymes represent a new evidence that exogenous F-1,6-P2 might represent an additional valid substrate for cell energy metabolism, of particular efficacy during period of metabolic sufferance characterized by mitochondrial dysfunction. Studies to determine the transport mechanisms involved in the cellular uptake of exogenous F-1,6-P2 are certainly needed.

## 4. Materials and Methods 

### 4.1. Organotypic Hippocampal Slice Cultures

The organotypic hippocampal slice cultures (OHSC) were prepared using a method described in detail elsewhere [34,58] and all procedures used were in accordance with UK regulations under the Animals (Scientific Procedures) Act of 1986 and approved by the Ethical Committee of the Catholic University of Rome (number 1F295.52, date 10-20-2017). Briefly, hippocampi from 8 to 10 days old Wistar rats were isolated and sliced at a thickness of 400 μm, using a McIlwain tissue chopper (Harvard Apparatus, Edenbridge, UK). The slices were then placed in plate inserts, 0.4 mm Millicel membrane and 30 mm diameter, previously treated with a coating solution of 320 μg/mL of poly-d-lysine (Sigma, St. Louis, MO, USA) and 80 μg/mL of laminin (Sigma) in sterile distilled water. The cultures were fed initially in 1200 μL NeuroBasal-A medium (Invitrogen, Loughborough, UK), containing B27 supplement (Invitrogen) (1 mL/100 mL medium), 5 mg/mL glucose (Sigma), 1 mM glutamine (Sigma) and incubated at 37 °C under humidified atmosphere of 95% O_2_ + 5% CO_2_. After two days in vitro, the NeuroBasal-A medium was replaced with full serum-containing medium, consisting of 25% heat-inactivated horse serum, 25% Hanks’-balanced salt solution, 50% minimum essential medium (all Invitrogen), 1 mM glutamine (Sigma), and 5 mg/mL D-glucose (Sigma). After approximately 10 days, when cultures adhered to the membrane and showed clear definition of the neuronal regions CA1, CA3 and dentate gyrus, fresh medium was added to the healthy cultures and the wells were used to perform the experiments.

### 4.2. NMDA and F-1,6-P2 Treatments

Excitotoxic effects of NMDA to OHSC were induced by adding 30 µM NMDA to culture medium for 3 h [59]. This NMDA concentration and timing of NMDA exposure were the best conditions causing significant (but not excessive) cell death and remarkable metabolic changes to OHSC, as evidenced by previous studies [59] and by preliminary experiments in which lower NMDA doses and/or shortest incubation times were tested (data not shown). At the end of this incubation time, the NMDA-containing medium was removed and OHSC were allowed to recover in fresh full serum-containing medium without or with the addition of F-1,6-P2 (0.5, 1 and 1.5 mM). The recovery time at 37 °C under humidified atmosphere of 95% air and 5% CO_2_ was prolonged for 24, 48 or 72 h. At each time point, the medium was collected and stored at −80 °C for LDH and lactate assays, the cultures were washed 3 times with 500 μL of ice-cold PBS and then processed to extract RNA for real-time quantitative PCR analysis. 

In a different set of experiments, using the same treatment protocol, cultures were washed 3 times with 500 μL of ice cold PBS and then immediately deproteinized with 500 μL of an ice cold organic solvent mixture (75% CH_3_CN + 25% 10 mM NaH_2_PO_4_, pH 7.4). This processing allowed obtaining samples suitable for the subsequent HPLC analysis of low molecular weight metabolites [60].

In a specific set of experiments, dedicated to evaluating the effects on gene expression, OHSC were incubated with F-1,6-P2 (1.5 mM) for 24, 48 and 72 h with no previous challenge with NMDA. Results obtained under the different experimental conditions were compared to OHSC incubated for the same times with neither previous NMDA challenge nor addition of F-1,6-P2. 

### 4.3. LDH and Lactate Assays

One hundred μL of each sample of culture medium from the various OHSC were used to determine LDH release as a measure of cell death. The enzyme activity was determined spectrophotometrically (Agilent 89090A, Agilent Technologies, Santa Clara Ca, CA, USA) following the time-dependent disappearance of NADH at 340 nm in a mixture containing 2 mM pyruvate, 200 μM NADH and 20 mM Tris-HCl buffer pH 7.6, according to standardized protocol [61].

The spectrophotometric determination of lactate was carried out following the method described by Artiss et al. [62]. Briefly, the reaction mixture contained 100 mM Tris-HCl, 1.5 mM *N*-ethyl-*N*-2-hydroxy-3-sulfopropyl-3-methylalanine, 1.7 mM 4-aminoantipirine, 5 IU horseradish peroxidase. One hundred µL of each sample were added to the mixture, let to stand for 5 min and read at 545 nm wavelength. The reaction was started with the addition of 5 IU of lactate oxidase to the cuvette (finale volume = 1 mL) and it was considered ended when no change in absorbance was recorded for at least 2 min. To calculate lactate, the difference in absorbance at 545 nm wavelength (Δabs) of each sample was interpolated with a calibration curve obtained by plotting Δabs measured in standard solutions of lactate with increasing known concentrations.

### 4.4. HPLC Analysis of Metabolites

The simultaneous separation of high-energy phosphates (ATP, ADP, AMP, GTP), purine nucleosides (inosine, adenosine, guanosine) and oxypurines (hypoxanthine, xanthine, uric acid) in the protein-free cell extracts (200 µL) was carried out using previously established ion pairing HPLC methods which utilize tetrabutylammonium hydroxide as the pairing reagent [63]. Separation was obtained using a Hypersil C-18, 250 × 4.6 mm, 5 µm particle size column, provided with its own guard column (Thermo Fisher Scientific, Rodano, Milan, Italy). The HPLC apparatus consisted of a SpectraSystem P4000 pump system (ThermoFisher Scientific) and a highly-sensitive UV6000LP diode array detector (ThermoFisher Scientific), equipped with 5 cm light path flow cell and set up between 200 and 300 nm wavelength. Assignment and calculations of the compounds of interest in chromatographic runs of cell extracts were performed at 260 nm wavelength by comparing retention times, absorption spectra, and area of the peaks of chromatographic runs of mixtures containing known concentrations of true ultrapure standard mixtures.

### 4.5. RNA Extraction and Real Time PCR Analysis

Total RNA was extracted from hippocampal slices using RNeasy® Mini Kit (QIAGEN, Manchester, UK) according to the instructions of the manufacturer. RNA was dissolved in RNase-free water and concentration and purity were determined with a ND-1000 UV–Vis Spectrophotometer (NanoDrop, ThermoFisher Scientidic, Waltham, MO, USA). RNA extract was reversed transcribed to cDNA by Superscript II Reverse Transcriptase Kit (Invitrogen). From each sample, 1 mg of total RNA, 500 ng of oligo dT primers (Roche Molecular Biochemicals, Burgess Hill, UK), 4 μL of First Strand Buffer and 200 U of Superscript II Reverse Transcriptase in a total volume of 20 μL were incubated at 42 °C for 50 min and 70 °C for 15 min.

Real Time PCR with melting curve analysis was performed in Bio-Rad iQ5 Real-time PCR Detection System (Bio-Rad, Hercules, CA, USA). In each reaction, 100 ng of cDNA sample was combined with a reaction mixture containing 25 μL of 2× SYBR Green PCR Master Mix (Applied Biosystems, Warrington, UK), each primer (at final concentration of 300 nM), and RNase-free water in a final volume of 50 μL. The thermal profile began with incubation at 95 °C for 10 min, followed by 40 cycles of amplification alternating between 94 °C for 15 s and 60 °C for 60 s. After amplification to confirm the specificity of reactions, a melting curve was produced by conducting 81 cycles of 30 s of melting every 0.5 °C from 55 to 95 °C. Final data were analyzed by the iQ™5 Optical System Software (Bio-Rad). Primers for PFKL (isoform L of 6-phosphofructo-1-kinase, NM_013190.4), ALDOC (aldolase C, NM_012497.1), GAPDH (glyceraldehyde-3-phosphate dehydrogenase, NM_017008.4) were designed with the 0.2 version of the Primer3 Input software developed by the Whitehead Institute for Biomedical Research (Cambridge, MA, USA) and using as template the sequences of *Rattus norvegicus*. For accurate gene expression measurements with Real Time PCR, results were normalized to a fixed reference, therefore the constitutively expressed housekeeping gene of *Rattus norvegicus* B2M (β-2-microglobulin, NM_017314.1) was selected using the geNorm Housekeeping Gene Selection Kit (Primer Design Ltd., Southampton, UK) from a list containing 12 candidate reference genes. Changes in transcript abundance of tested genes were calculated using the 2^−ΔΔ*C*T^ method as described by Livak and Schmittgen [64].

### 4.6. Statistics

The within group comparison at each time was performed by the one-way analysis of variance (ANOVA). Differences across groups were estimated by the two-way ANOVA. Fisher’s protected least square was used as the post hoc test. Only *p*-values of less than 0.05 were considered as statistically significant.

## Figures and Tables

**Table 1:** Relative expression of genes encoding for the glycolytic enzymes 6-phosphofructo-1-kinase (PFKL), aldolase (ALDOC) and glyceraldehyde-3-phosphate dehydrogenase (GAPDH) in OHSC incubated for different times in presence of increasing concentrations of F-1,6-P2.

Treatment	Time (Hours)	PFKL/BD2M (Fold Increase)	ALDOC/BD2M (Fold Increase)	GAPDH/BD2M (Fold Increase)
**Control**	24	1.10 ± 0.30	1.02 ± 0.18	1.15 ± 0.13
	48	1.07 ± 0.26	1.15 ± 0.21	1.07 ± 0.20
	72	0.98 ± 0.21	1.08 ± 0.23	0.99 ± 0.14
**0.5 mM F-1,6-P2**	24	4.64 ± 0.62 ^a,b^	0.94 ± 0.11	1.14 ± 0.25
	48	7.89 ± 0.88 ^a,b^	1.09 ± 0.18	1.09 ± 0.18
	72	11.26 ± 1.23 ^a,b^	3.67 ± 0.43 ^a,b^	8.38 ± 1.23 ^a,b^
**1 mM F-1,6-P2**	24	5.16 ± 0.49 ^a,b^	1.11 ± 0.17	1.06 ± 0.16
	48	10.54 ± 1.18 ^a,b^	0.97 ± 0.09	0.94 ± 0.10
	72	15.92 ± 1.66 ^a,b^	4.71 ± 0.75 ^a,b^	11.55 ± 2.03 ^a,b^
**1.5 mM F-1,6-P2**	24	9.75 ± 0.95 ^a,b^	1.21 ± 0.26	1.19 ± 0.21
	48	14.21 ± 1.58 ^a,b^	1.34 ± 0.31	1.32 ± 0.18
	72	21.09 ± 2.36 ^a,b^	6.92 ± 0.95 ^a,b^	17.46 ± 2.67 ^a,b^

Values are the mean ± S.D. of 5 experiments from 5 different OHSC preparations and are expressed as fold increase respect to values recorded in control OHSC at corresponding times (see Figure 7 for control values of PFKL and Figure 8 for those of ALDOC and GAPDH). ^a^ Significantly different from controls, *p* < 0.001. ^b^ Significantly different from values recorded in OHSC previously challenged with 30 μM NMDA and then supplemented with the same F-1,6-P2 concentration, *p* < 0.01.
